# Life‐history attributes of juvenile *Anopheles gambiae* s.s. in central Uganda; implications for malaria control interventions

**DOI:** 10.1111/mve.12568

**Published:** 2022-04-07

**Authors:** Charles Batume, Anne M. Akol, Louis G. Mukwaya, Josephine Birungi, Jonathan K. Kayondo

**Affiliations:** ^1^ Department of Entomology Uganda Virus Research Institute (UVRI) Entebbe Uganda; ^2^ Department of Zoology, Entomology and Fisheries Sciences College of Natural Sciences, Makerere University Kampala Kampala Uganda; ^3^ ILRI‐ Biosciences International Livestock Research Institute Nairobi Kenya

**Keywords:** entomopathogens, genetic modification, larvicides, vector bionomics

## Abstract

Malaria is among the leading causes of death in Uganda, and *Anopheles gambiae* sensu stricto (s.s.) is the predominant vector. Although current vector control interventions have greatly reduced the malaria burden, the disease persists. New interventions are needed in order to eradicate them. Evaluation of new tools will require the availability of well‐characterized test vector populations. Juvenile *An. gambiae* s.s. from Kibbuye and Kayonjo‐derived populations were characterized under semi‐field and laboratory conditions, given that various vector traits, including abundance and fitness are dependent on development profiles at this life stage. Ten replicates comprising 30 first instar larvae each were profiled for various life‐history attributes (egg hatching, larval development time, larval survivorship, pupal weight and pupation rate). All parameters were similar for the two sites under laboratory conditions. However, the similarities or differences between field and laboratory development were parameter‐specific. Whereas, larval survivorship and pupal weight were similar across seasons and laboratory in colonies from both sites, in the semi‐field settings, pupation rate and larval survivorship differed between seasons in both sites. In addition, the average larval development time during the wet season was longer than that of the laboratory for both sites. Availability of mirror field sites is important for future tool evaluations.

## Graphical abstract

Experimental replicates as setup in the laboratory (A) and the field (B)

- (a) Replicates in laboratory experimental setup. (b) Replicates in field experimental setup.
- All parameters were similar for the two juvenile *Anopheles gambiae* s.s. populations under laboratory conditions.
- Larval survivorship and pupal weight were similar in dry and wet seasons, and in the laboratory, for both sites.
- The average larval development time during the wet season was longer than that laboratory for both sites.

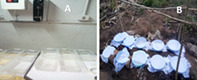

## INTRODUCTION

Malaria, a mosquito vector‐borne disease, is a leading cause of morbidity and mortality in sub‐Saharan Africa. The African region contributes over 94% of global cases, in 2019, Uganda alone contributed about 5% of the global cases (WHO, 2020). More than 95% of the country lies in high malaria transmission areas. Malaria accounts for over 20% of hospital outpatient visits and up to 19% of inpatient admissions (President's Malaria Initiative, 2020), thereby imposing a huge burden on the country's healthcare system. Current malaria control interventions rely on indoor residual spraying of insecticides, usage of insecticide‐treated bed nets and drug therapy. However, in spite of the control scale ups made over the past decade, malaria persists. *Anopheles gambiae* sensu stricto (s.s.) is the principal vector of malaria in Uganda, while *Anopheles funestus* and *Anopheles arabiensis* are considered secondary vectors (Presidential Malaria Initiative, 2016). The progress in the fight against malaria has slowed significantly since 2015, with malaria vector and parasite counter adaptations to mainstream control measures and budget shortfalls being among the challenges contributing to malaria persistence (Guyant et al., 2015). There is, therefore, a need to develop additional interventions to supplement current malaria control efforts if we are to eliminate the disease.

Mosquito population size can vary greatly depending on several larval development and growth factors (Barreaux, 2018). Knowledge of life‐history parameters of vector development attributes such as survivorship, size and growth rates among others provides important data for site characterization and modelling to predict the impact of control interventions. Although most malaria control efforts are targeting the adult stage, programmes that target aquatic immature stages are increasingly gaining interest to supplement the core indoor insecticide‐based interventions (Derua et al., 2019). This is because the vector capacity, the intensity of transmission and fitness of adult mosquitoes are dependent on juvenile stages. Larviciding is one of the control efforts that will benefit from enhanced understanding of site‐specific juvenile life‐history attributes of target vector populations. For example, survivorship and development time of mosquito stages can help determine when to apply a control intervention in a given site. Malaria elimination has not been achieved despite decades of control efforts, and so innovative approaches that complement current methods are needed.

*Anopheles gambiae* s.s. juvenile stages development data exists but only from a handful of locations in Africa. In Burkina Faso, there were notable differences in terms of the phenotypic and physiological development of larvae reared in the insectary compared to semi‐field conditions (Mouline et al., 2012). In Tanzania, Eliningaya et al. (2005) observed that the development and survival of mosquito larvae were higher in semi‐field conditions than in the insectary. However, in the same study, Eliningaya et al. (2005), obtained similar pupation and adult emergence rates in the insectary and semi‐field conditions. This was attributed to differences in light intensity and temperatures in the field compared to the insectary.

Mosquito life‐history attributes in the East African region are expected to vary given the diversity in the climate, physical features and ecology. The region experiences the largest inter‐annual rainfall variations in the world, although a drying trend in March–May rainy season has been observed since the 1980s. The strong, sometimes non‐linear altitudinal gradients of temperature and moisture regimes, also contribute to the climate diversity of Eastern Africa (Camberlin, 2018). These variations in climatic and environmental conditions in the region may result in the adaptation of mosquito species, leading to changes in species composition and development traits and subsequent changes in the dynamics of mosquito‐borne disease transmission (Afrane et al., 2012).

Life‐history attributes of juvenile *An. gambiae* s.s. in Uganda are poorly described. Most studies have focused on the field ecology and behaviour of adult mosquitoes (Mutebi et al., 2014; White, 2008). To our knowledge, the only available data on life‐history attributes of mosquitoes in immature stages comes from a few studies done on *Aedes* species in Uganda (Lutwama & Mukwaya, 1995; Sempala, 1981). The knowledge of life‐history attributes of *An. gambiae* s.s. in Uganda is, therefore, warranted. In this study, we measured larval developmental time, larval mortality, pupae weight and rate of pupation under laboratory and semi‐field conditions in two *An. gambiae* mosquito populations in Uganda. Well‐characterized natural populations could provide ideal test sites for future evaluation of the effectiveness of various vector‐based control measures. Insights into the ecological, environmental and/or biological differences at the aquatic stage in nature could be additionally harnessed for malaria control.

## MATERIALS AND METHODS

### Study area

**FIGURE 1:**
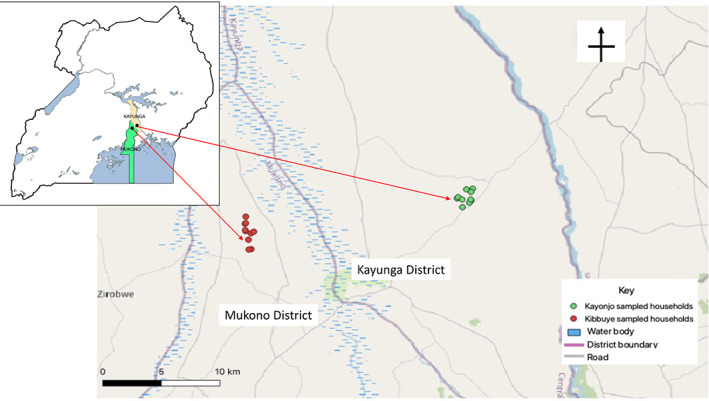
A map of Kayunga and Mukono Districts showing households where Mosquito samples were collected

Mosquitoes were collected from Kibbuye Village in Mukono District (0.2835°N, 32.7633°E) and Kayonjo Village in Kayunga District (0.9860°N, 32.8536°E) in Central Uganda (Figure 1). Both districts experience two rainy seasons and two dry seasons per year. The first rainy season is generally from March to June, followed by a dry season from July to September. The second shorter rainy season runs from October to November and is followed by a dry period from December to February. The districts experience an average annual rainfall of about 1435 mm (Data‐Africa, Uganda Mukono/ Kayunga, 2015). Temperatures typically vary from 16.7°C to 27.8°C, although the water temperatures of mosquito breeding habitats could be a degree higher or lower than the environmental temperature. The study sites have rich flora that include forest and swamp vegetation, savannah short grasses and thorny bushes. One village was selected from each district based on several factors, including human settlements, vegetation type, the prevalence of *An. gambiae* s.s. mosquitoes and malaria endemicity. The two districts experience high malaria incidences (up to 150 confirmed malaria cases per 1000 population/year) and are located in areas of high mosquito densities of *An gambiae* s.s. (Ministry of Health, 2014). The selected districts have reportedly high levels of insecticide resistance associated with knockdown resistance (*kdr*) mutation *Vgsc*‐L1014S in *An. gambiae* s.s. (Lynd et al., 2019). Ethical approval was obtained from Uganda Virus Research Institute Ethics Committee (GC/127/16/11/348) and the Uganda Council for Science and Technology (HS 1328). Informed consent was obtained from the sub‐county leaders, village leaders and household owners before gravid females were collected.

### Field collections and processing

**FIGURE 2:**
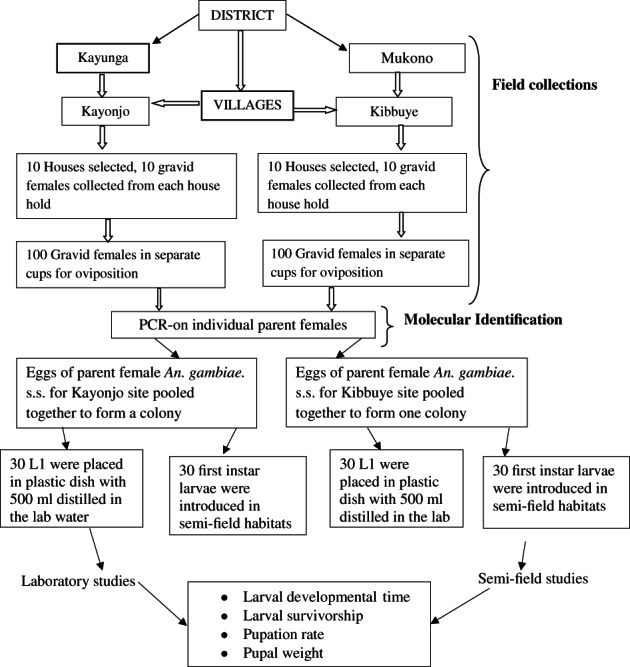
The schematic of sampling protocol and experiments

Laboratory and semi‐field‐reared samples were used to generate various population‐level mosquito development life attributes (Figure 2).

### Indoor collection of adult mosquitoes

Ten houses from each study village were randomly selected for indoor collections of adult mosquitoes in each district. Collections were made at the end of the rainy season in January 2017 (Figure 1). The collections were used to establish mosquito colonies as a first step for the study (Figure 2). Informed consent to sample from the village was obtained from sub‐county leaders, village leaders and household owners, respectively, before embarking on the collection of gravid females. Collections were made in the morning hours from 06:00 to 09:00 AM. Coordinates of the selected houses were recorded using a handheld global positioning device (Garmin GPSMAP® 64s, Garmin. Olathe, Kansas, USA). Ten gravid females identified using morphological keys (Gillies & Coetzee, 1987) as *An. gambiae* were collected from each house using a mouth aspirator. Mosquito samples collected from each house were individually gently placed into separate 250 ml paper cups fitted with a net at the top as previously described (Coluzzi & World Health Organization, 1973). The cups were placed in a cage and immediately transported to the insectary at Uganda Virus Research Institute, Entebbe, Uganda for further rearing. The gravid females in the insectary were fed on 10% glucose for 3–4 days to attain full egg development. A forced‐egg laying method was used to induce the females to lay eggs (Morgan et al., 2010). Each female that oviposited was killed by freezing at −80°C for 2 min, transferred into a labelled tube containing 80% alcohol and stored at −80°C.

### Molecular species identification of mosquitoes

Each field‐caught female (F_o_) that oviposited first generation (F_1_) egg batches were subjected to species diagnostic Polymerase Chain Reaction (PCR)‐analysis for molecular species identity confirmation as described by Wilkins et al. (2006).

### PCR amplification

Two legs, as DNA template from each mosquito, were directly dropped in an aliquot of PCR reaction mix consisting of; 1 U of Taq DNA polymerase (Invitrogen), 0.3 mM MgCl_2_, 0.08 mM dNTPs, 1 μM of each primer (Wilkins et al., 2006) and buffer (Invitrogen, Life Technologies corp. Carlsbad, CA, USA) at 1× concentration in a distilled water (dH_2_0) topped‐up 25 μl reaction volume. Primers (Eurofins genomics) consisted of Universal IMP‐UN as forward primer and respective reverse primers; ME‐3T for *An. merus*, QD‐3T for *An. quadriannulatus*, GA‐3T for *An. gambiae* and AR‐3T for *An. arabiensis* for potential *An. gambiae* candidate sibling species prevalent in the region. The PTC‐100^MT^ thermocycler (MJ Research Inc, Watertown, MA, USA) was used for amplification following the Wilkins et al. (2006) protocol. PCR products were separated by electrophoresis through 1% agarose gels and visualized by ultraviolet illumination after gel staining with ethidium bromide.

### Colony establishment

Following molecular species identification of F_o_ adult females, oviposited eggs from confirmed *An. gambiae* s.s. were used to establish the colonies (Figure 2). The colonies were reared following protocols described by Benedict (2007). Two colony lines (one for each site) were established for Kayonjo and Kibbuye sites. The F_1_ colonies were used to rear mosquitoes for life‐history investigations. Emerging larvae were placed in plastic dishes (30 cm × 15 cm × 15 cm size) containing 500 ml of dH_2_o. Larvae were reared at temperature ranges of 24°C–28°C. The larvae were given a daily portion of 10 mg/day of fish food (Tetramin® Germany, Teyra GmbH Company) as previously described by Bayoh and Lindsay (2004), Kirby and Lindsay (2009), Bock et al. (2015). Emerging adult mosquitoes were maintained in 30 cm × 30 cm × 30 cm holding cages at 60%–70% relative humidity at 24°C–28°C. The adults were fed on 10% glucose solution from soaked cotton pads. The colonies were maintained up to the 6th generation (F_6_) before the commencement of the life‐history attribute studies. At the F_6_ generation, egg production and mosquito survivorship had become similar between subsequent generations by that stage indicating colony stability. The purity of the colony as *An. gambiae* s.s. was ascertained by molecular identification of a sample of 10 dead males and females from each generation.

### Experimental set up in the insectary

A total of 30 first instar larvae of *An. gambiae* s.s., obtained within 3 h of hatching from the colonies, were used in replicates of 10 for each study site. The number of larvae used was similar to that used by Bayoh and Lindsay (2003). The rearing procedure for colony establishment was as described above. The rearing water was changed every 2 days by transferring the larvae from one dish into another with fresh distilled water. The temperature of water in the rearing dishes was maintained at 26 ± 1°C in a thermostat‐controlled room. The relative humidity varied between 64% and 70% as measured by a hygrometer. The larval dishes were inspected daily every 6 h and dead larvae were removed using a pipette, counted and recorded. The number of individuals transforming into the next larval stage was recorded daily until pupation. Emerging mosquitoes that died were recorded as part of pupal mortality.

### Determination of life‐history attributes of juveniles

Percentage of eggs hatching: This was determined as the number of eggs that hatched out of the total number of eggs that were placed in a dish for hatching expressed as a percentage. Larval survivorship: This was determined as the number of fourth instar larva that developed into pupae stage out of initial first instar cohort in all replicates (300). Larval development time: This was determined as the average time spent for larvae to develop from the first instar stage to pupae. The stage duration was determined when 50% of individuals in one stage had transformed into the next immature stage (Bayoh & Lindsay, 2003). Pupal weight was recorded as the average weight of the pupae in each of the 10 replicates. The weight was measured using an electronic balance (model Mettler PE 200) to the nearest 0.01 mg. Pupation rates were recorded as the total number of pupae collected per time (measured in days) taken to develop from the fourth instar larva to the pupation.

### Semi‐field experiment setup

Semi‐field life‐history experiments were set up at Kibbuye and Kayonjo study sites during both the dry and wet seasons. Dry season studies ran from July to September 2017. Ten semi‐field habitats were made using plastic washbowls (35 cm diameter × 20 cm deep). In the bowls, 3 kg of soil from the study site was mixed with 3 L of screen filtered pond water to form mud. Pond water used as semi‐field habitats was obtained from one large pond in each study site that contained *Anopheles* mosquitoes. The mud was left to dry to mimic the natural soil lining of habitats. The method used for setting up semi‐field habitats is a modification of Gimnig et al. (2002) in that there was no variation in the larval density in the replicates and the larvae were not given food supplements. The larvae fed on algal biomass found in the artificial habitats. The pond water was filtered using screens and topped‐up daily to replenish the amount lost due to evaporation. Ten semi‐field habitats were set up for each site and left to stand for 6 weeks before the introduction of larvae. Each washbowl was covered with insect netting to prevent other mosquitoes from ovipositing in the artificial habitats. *An. gambiae* s.s. larva hatched from the established colony in the insectary were transferred to the field site. A total of 30 first instar larvae (within 6 h after hatching) were introduced into the artificial habitats in the field. The field life‐history attributes were recorded and captured as earlier described in the laboratory. In addition, the mean physio‐chemical conditions of water in semi‐field habitats were measured daily for pH and conductivity using a standard portable pH–conductivity, total dissolved solids and temperature combination metre (Hannainstruments.co.uk) from the first stage to adult emergence. For the control experiments, 10 artificial habitats were set up that were not seeded with larvae. The habitats were protected from rain by installing an overhead sheet 2 m above the ground as a protective cover. The same experimental setup was repeated for the wet season experiments that ran from September to December 2017.

### Data analysis

The life‐history characteristics, larval development time, larval survivorship, pupal weight and pupation rate were analysed for larvae reared under laboratory conditions and field conditions. Data were tested for normality using the Shapiro Wilks normality test before further analysis. Comparison of means of life‐history attributes between seasons and sites was also performed using multivariate analysis of variance (MANOVA). Multivariate analysis was used since the life‐history attributes were measured at once for the same population. The water physio‐chemical parameters were measured for their correlation with life‐history attributes using Pearson correlation. All analyses were done using the SPSS‐statistical package (version 16.0 for windows SPSS Inc., Chicago, IL, U.S.A.).

## RESULTS

### *Life‐history attributes of immature* An. gambiae *s.s. from Kayonjo colony*

#### Laboratory studies

Out of the 542 eggs placed in the dishes, 67 ± 2.2% of them hatched. Three hundred first instar larvae derived from the hatched eggs were used for the 10 replicates. Stage‐specific survivorship rates were over 90% in all larval stages, and 88% at the pupae stage. The mean duration of the specific larval stages varied; the least was 1.2 ± 0.15 days in the third instar and the highest was 2.3 ± 0.2 days in the second instar. The mean pupae duration was 1.4 ± 2.6 days. Pupation rate and mean pupae weight were 14.6 ± 2.6 pupae per hour and 4 ± 0.2 mg, respectively. On average, it took 8.4 ± 0.4 days (7.7–8.9 days) for *An. gambiae* s.s. from egg hatching to adult emergence (Table 1).

**TABLE 1:** Life‐history attributes of *Anopheles gambiae* s.s. immature stages reared in laboratory and field for Kayonjo site

	Hatching rate	Larval development time in days	Number of immature surviving per stage	Larval survivorship %	Average pupal weight	Pupation rate
Kayonjo laboratory						
Egg	67.0 ± 2.2					
First instar		1.8 ± 0.4	289	96.3		
Second instar		2.3 ± 0.3	263	87.7		
Third instar		1.2 ± 0.1	249	83.0		
Fourth instar		1.7 ± 0.3	229	76.3		
Pupae		1.4 ± 0.2	201	67.0	4.0 ± 0.2	14.6 ± 2.6
First instar‐adult		8.4 ± 0.4	201	82.1		
Kayonjo dry season						
Egg	69.4 ± 1.6					
First instar		1.7 ± 1.1	286	95.3		
Second instar		1.7 ± 0.2	272	95.1		
Third instar		1.5 ± 0.1	271	99.6		
Fourth instar		1.6 ± 0.1	254	93.7		
Pupae		1.1 ± 0.1	217	85.4	3.0 ± 0.8	15.3 ± 2.6
First instar‐adult		7.6 ± 0.3	217	72.3		
Kayonjo wet season						
Egg	73.4 ± 1.3					
First instar		1.45 ± 0.08	262	87.3		
Second instar		2.29 ± 0.09	249	83.0		
Third instar		1.5 ± 0.16	245	81.7		
Fourth instar		2.1 ± 0.11	218	72.7		
Pupae		0.94 ± 0.11	193	64.3	3 ± 0.001	15.0 ± 2.4
First instar‐adult		8.32 ± 0.17	193	77.8		

*Note*: Starting first instar larvae (*n* = 300). Survival rate (*S*_i_) was determined according to the formula: *S*_i_ = *n*_i_/(*xn*_i_ – 1) × 100; where, *n*_i_ is the number of larvae entering instar i, and *xn*_i_ – 1 is the number of larvae that entered the preceding instar.

#### Field studies during the dry season

The percentage of egg hatching from the insectary parent stock used for dry season artificial habitat seeding was 69 ± 1.6% out of the 640 eggs. Of the hatched eggs, 300 first instar larvae, were used in the artificial habitats. The mean duration of the specific larval stages varied; the least was 1.5 ± 1.1 days in the third stage and the highest was 1.7 days in the second and first instar. On average, it took 7.6 ± 0.2 days for *An. gambiae* s.s. to hatch from egg to adult. The pupation rate and pupae weight were 15.3 ± 2.6 and 3 ± 0.8 mg, respectively. The mean survival rate was 85.4% at the pupae stage and the overall mean survival rate from egg hatching to adult emergence was 72.3% (Table 2). The habitat temperatures at night ranged from 17.5°C to 23°C and 19°C to 32°C during the day (Table 3).

**TABLE 2:** Life history attributes of *Anopheles gambiae* s.s. immature stages reared in the laboratory and field for the Kibbuye site

	Hatching Rate	Larval development time in days	Number of immature surviving per stage	Larval survivorship %	Average pupal weight	Pupation rate
Kibbuye laboratory						
Egg	58.0 ± 2.6					
First instar		1.8 ± 0.1	294	98.0		
Second instar		2.0 ± 0.3	282	94.0		
Third instar		1.1 ± 0.2	273	91.0		
Fourth instar		1.6 ± 0.3	244	81.3		
Pupae		1.5 ± 0.2	205	68.3	4.0 ± 1.6	15.2 ± 2.8
First instar‐adult		8.1 ± 0.4	205	86.5		
Kibbuye dry season						
Egg	61 0.13 ± 2.3					
First instar		2.1 ± 0.1	282	94		
Second instar		2.1 ± 0.1	263	93.3		
Third instar		1.3 ± 0.3	258	98.09		
Fourth instar		2.3 ± 0.1	219	84.8		
Pupae		1.1 ± 0.1	184	84.01	3.6 ± 0.8	9.76 ± 1.18
First instar‐adult		8.9 ± 0.5	184	61.3		
Kibbuye wet season						
Egg	63.6 ± 1.9					
First instar		1.6 ± 0.07	232	77.3		
Second instar		2.4 ± 0.15	190	63.3		
Third instar		1.36 ± 0.15	178	59.3		
Fourth instar		2.2 ± 0.23	156	52.0		
Pupae		1.02 ± 0.13	145	48.3	3.0 ± 3	5.2 ± 1.58
First instar‐adult		8.69 ± 0.2	145	60.3		

**TABLE 3:** Physico‐chemical conditions of water in semi‐field habitats in Kibbuye and Kayonjo study site for wet season and dry season

Parameter	Kayonjo		Kibbuye	
	Wet season	Dry season	Wet season	Dry season
Total dissolved solids/pmm	30.67 ± 0.17	35.18 ± 0.11	32.7 ± 0.42	38 ± 0.32
Conductivity/μmhos cm	43.56 ± 0.13	83.34 ± 0.14	54.56 ± 1.3	76.14 ± 1.4
pH	7.8	7.3	7.2	7.7
Average temperature	22.7°C	23.0°C	19.1°C	20.6°C
Relative humidity	82.90%	79.10%	86.20%	84.10%

### Field studies during the wet season

The mean duration of the larval stages varied; the least was 0.94 ± 0.11 days in the pupal stage and the highest was 2.29 ± 0.09 days in the second instar. Pupation rate and mean pupae weight were 15.0 ± 2.4 pupae per hour and 3 ± 0.001 mg, respectively. On average, it took 8.32 ± 0.17 days for *An. gambiae* s.s. from egg hatching to adult emergence (Table 1).

### Comparison between dry and wet seasons for Kayonjo site

Overall, significant differences in life‐history attributes were observed between field mosquito development aspects during wet and dry season at Kayonjo study sites (*F*_(4,11)_ = 24.376, *p* = 0.001; Wilk's Λ = 0.101 at 95% confidence interval). Larval development time was significantly longer during the wet season (*F*_(1,14)_ = 59.644, *p* = 0.001) than in the dry season. The pupation rate was significantly higher in the dry season (*F*_(1,14)_ = 21.943, *p* = 0.001) as compared to the wet season. Larval survivorship was significantly higher during the dry (*F*_(1,14)_ = 9.00, *p* = 0.01) than wet season. However, there was no significant difference at Kayonjo in pupal weight between seasons (*F*_(1,14)_ = 1.149, *p* = 0.303).

### Comparison of life‐history attributes between laboratory and semi‐field experiments for Kayonjo dry season

The development time of larvae reared under semi‐field conditions in the dry season at Kayonjo was significantly shorter (*F*_(1,18)_ = 7.603, *p* = 0.013) (6.487 ± 0.29 days) than in the laboratory (6.96 ± 0.46 days). However, there was no significant difference between larvae reared in laboratory and field conditions in pupation rate (*F*_(1,18)_ = 0.349, *p* = 0.562), larval survivorship (*F*_(1,18)_ = 0.3, *p* = 0.975) and pupal weight (*F*_(1,18)_ = 3.955, *p* = 0.337).

### Comparison of life‐history attributes between laboratory and semi‐field conditions for Kayonjo site wet season

There was a statistically significant difference in some life‐history attributes between laboratory and field‐reared mosquitoes from Kayonjo study site in the wet season (*F*_(4,15)_ = 3.056, *p* = 0.003; Wilk's Λ = 0.366 at 95% confidence interval). Larval development time was significantly longer (*F*_(1,18)_ = 7.757, *p* = 0.012), (7.4 ± 0.3 days) under field conditions compared to laboratory conditions (6.9 ± 0.5 days). Pupation rate of larvae was significantly higher (*F*_(1,18)_ = 24.26, *p* = 0.0001), for larvae reared under the laboratory compared to field conditions. However, there was no significant difference in larval survivorship and pupae weight (*F*_(1,18)_ = 2.179, *p* = 0.157) and (*F*_(1,18)_ = 1.426, *p* = 0.248), respectively, for larvae reared under laboratory and field conditions.

### *Life‐history attributes of immature* An. gambiae *s.s. from Kibbuye colony*

In the laboratory, out of the 614 eggs of the Kibbuye colony that were placed in the dish, 58 ± 2.6% eggs hatched. The total number of larvae used in the 10 replicates was 300. On average, it took 8.14 days (7.7–8.7 days) for *An. gambiae* s.s. to emerge from egg to adult. Stage‐specific survivorship was over 90% in all larval stages, and 84% for the pupal stage. Pupation rate and pupal weight were 15.2 ± 2.8 and 4 ± 1.6 mg, respectively. The mean pupal duration was about 1.5 days. The overall mean survival rate from egg hatching to adult emergence was 68.3% (Table 2).

### Field studies during the dry season

The percentage of eggs hatched was 61 ± 2.3% out of the 720 eggs from the insectary parent stock. The mean duration of the specific larval stages varied in the dry season; the least was 1.1 ± 0.1 days in the pupal stage and the highest was 2.3 ± 0.1 days in the fourth instar. The pupal stage lasted for a mean period of 1.2 ± 0.14 days. On average, it took 8.9 ± 0.5 days for *An. gambiae* s.s. to develop from egg to adult. Pupation rate and pupae weight was 9.7 ± 1.2 and 3.6 ± 0.8 mg, respectively. The mean survival rate was 84% for the pupae stage and the overall mean survival rate from egg to adult emergence was 61% (Table 2). Semi‐field habitat water temperature averaged at about 20.6°C, with the temperatures of habitat at night ranging from 12.5°C to 24.5°C and 15°C to 30.5°C during the day (Table 3).

### Field studies during the wet season

The overall mean survival rate from egg to adult emergence was 60.3% from the Kibbuye stock in the wet season. The mean duration of the specific larval stages varied; the least was 1.02 ± 0.13 days in the pupal stage and the highest was 2.4 ± 0.157 days in the second instar. Pupation rate and mean pupae weight were 5.2 ± 1.58 pupae per hour and 3.0 ± 3 mg, respectively. On average, it took 8.69 ± 0.21 days for *An. gambiae* s.s. from egg hatching to adult emergence (Table 2).

### Comparison of attributes between wet and dry season in Kibbuye site

There was a statistically significant difference in some life‐history attributes between field experiments conducted during the wet and dry season at the Kibbuye study site (*F*_(4,11)_ = 8.509, *p* = 0.02; Wilk's Λ = 0.244 at 95% confidence interval). The pupation rate was significantly higher (*F*_(1,14)_ = 38.581, *p* = 0.001) in the dry season compared to the wet season. Larval survivorship was significantly higher (*F*_(1,14)_ = 27.75, *p* = 0.001) in the dry than in the wet season. The rest of the development parameters, including pupal weight (*F*_(1,14)_ = 2.133, *p* = 0.166) and larval development time (*F*_(1,14)_ = 0.001, *p* = 0.98) were not significantly different between the wet and dry season.

### Comparison of attributes between laboratory and dry season in Kibbuye site

**FIGURE 3:**
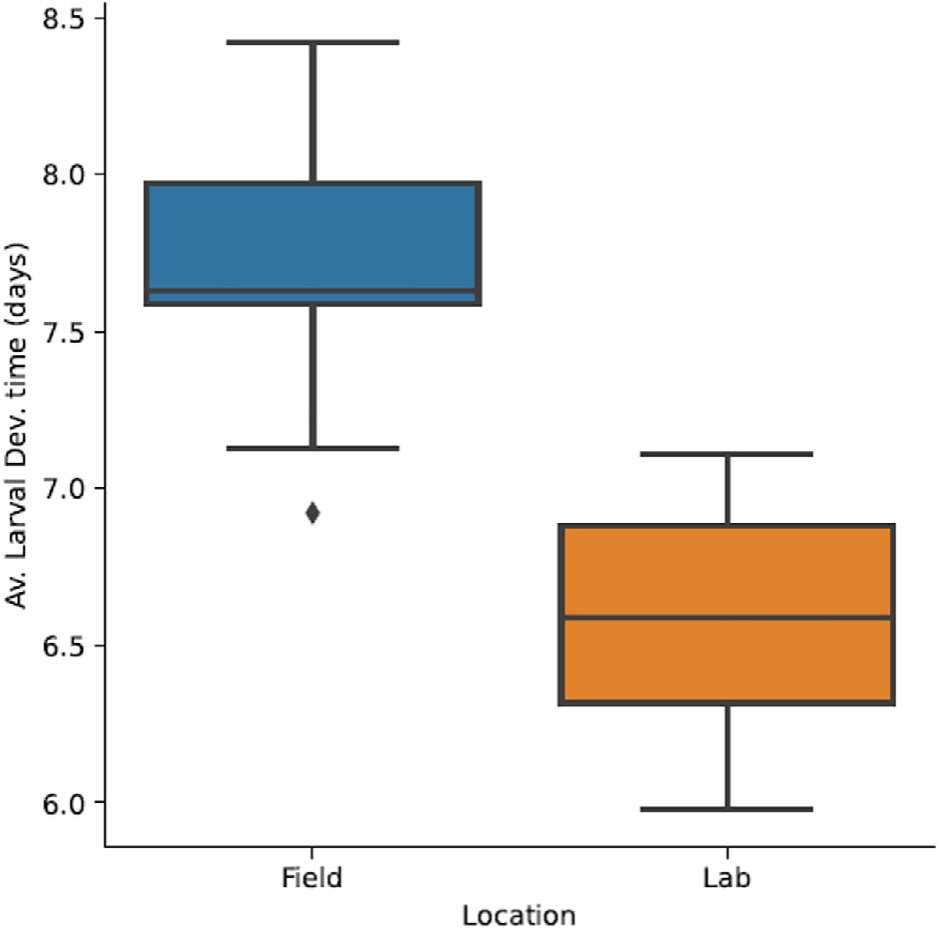
Comparison of larval development time under laboratory and field for Kibbuye site dry season

There was a statistically significant difference in some life‐history attributes between laboratory and field‐reared mosquitoes from Kibbuye in the dry season (*F*_(4,15)_ = 16.037, *p* = 0.001; Wilk's Λ = 0.19 at 95% confidence interval). Development time of larvae was significantly shorter (*F*_(1,18)_ = 35.053, *p* = 0.001), (6.59 ± 0.4 days) for larvae reared under laboratory conditions compared to 7.7 ± 0.46 days under field conditions (Figure 3). The pupation rate of larvae reared under laboratory conditions was significantly higher (15.19 ± 0.901) compared to the field ((9.76 ± 0.37); (*F*_(1,18)_ = 30.97, *p* = 0.001). However, there was no significant difference in larval survivorship and pupae weight under laboratory conditions compared to field conditions (*F*_(1,18)_ = 2.83, *p* = 0.11) and (*F*_(1,18)_ = 0.36, *p* = 0.554), respectively.

### Comparison of life‐history attributes between laboratory and wet season in Kibbuye site

There was a statistically significant difference in some life‐history attributes between laboratory and field‐reared mosquitoes from Kibbuye in the wet season (*F*_(4,15)_ = 3.056, *p* = 0.0001; Wilk's Λ = 0.025 at 95% confidence interval). Larval development time was significantly longer (7.6 ± 0.4 days; *F*_(1,18)_ = 58.269, *p* = 0.0001) for larvae reared under field compared to laboratory conditions (6.6 ± 0.5 days). Pupation rate was significantly higher (*F*_(1,18)_ = 168.34, *p* = 0.0001) for larvae reared under laboratory conditions compared to the field conditions. However, there was no significant difference in larval survivorship and pupae weight (*F*_(1,18)_ = 3.16, *p* = 0.092) and (*F*_(1,18)_ = 1.305, *p* = 0.268), respectively, for larvae reared under laboratory and field conditions.

### Water parameters between sites and across seasons

Generally, the water parameters had limited or no effect at all on the life‐history attributes between sites and across seasons. Conductivity had a weak positive correlation on pupation rate (*r* = 0.279, *p* > 0.05) and larval survivorship (*r* = 0.287, *p* > 0.05) during the dry season in Kayonjo site. Conductivity did not affect pupal weight and larval development time between site and seasons. There was also a weak positive correlation of pH on life‐history attributes in the two sites and seasons. Relative humidity showed a weak positive correlation in the wet season and no effect in the dry season. Total dissolved solids showed no correlation with life‐history attributes in the two sites and seasons (Table 3).

### Comparisons between sites

#### Comparison of growth parameters between sites under laboratory conditions

Under laboratory conditions, there were no significant differences between Kayonjo and Kibbuye sites (*F*_(4,15)_ = 0.99, *p* = 0.438; Wilk's Λ = 0.79 at 95% confidence interval) in all growth parameters, that is, hatch rate, larval survivorship, pupation rate, pupal weight and larval developmental time of the juvenile stages.

#### Comparison of growth parameters during the dry season between sites

Larval development time at the Kibbuye site was significantly longer than that of the Kayonjo site during the dry season (*F*_(1,18)_ = 50.050, *p* = 0.001). Pupation rate for the Kibbuye site was significantly lower (*F*_(1,18)_ = 37.98, *p* = 0.001) than that of the Kayonjo study site; and larval survivorship for the Kibbuye site was significantly lower (*F*_(1,18)_ = 10.72, *p* = 0.004) than that at Kayonjo site. However, pupal weight (*F*_(1,18)_ = 2.28, *p* = 0.148) was not significantly different in the two study sites under field conditions in the dry season.

#### Comparison of growth parameters during the wet season between sites

**FIGURE 4:**
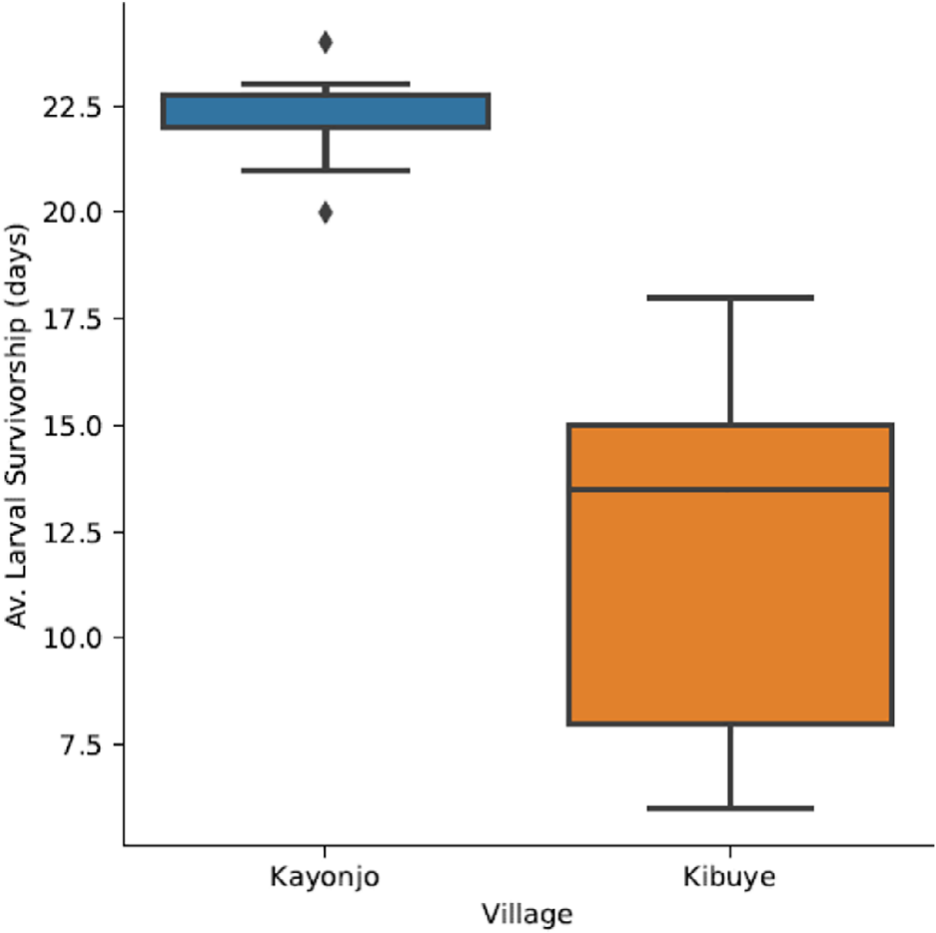
Comparison of wet season larval survivorship between Kayonjo and Kibbuye sites

There was statistically significant difference in some life‐history attributes between sites during the wet season (*F*_(4,11)_ = 19.309, *p* = 0.001; Wilk's Λ = 0.125 at 95% confidence interval). Larval development time at the Kayonjo site was significantly shorter (*F*_(1,14)_ = 5.947, *p* = 0.029) than that in the Kibbuye site. In addition, the pupation rate in the Kibbuye site was significantly lower (*F*_(1,14)_ = 64.109, *p* = 0.001) as compared to that in the Kayonjo site. Larval survivorship in the Kayonjo site was significantly higher (*F*_(1,14)_ = 31.461, *p* = 0.029) than that in the Kibbuye site during the wet season (Figure 4). Pupal weight (*F*_(1,14)_ = 0.192, *p* = 0.668) was not significantly different between sites.

## DISCUSSION

The life‐history attributes of immature *An. gambiae* s.s. mosquitoes in Uganda were investigated and their profiles across sites and seasons under laboratory and field settings described. This study was important in generating baseline data that could aid the design and evaluation of control interventions. The study revealed that hatching rates, larval development times, pupation rate, larval survivorship and pupal weight were not significantly different between the two *An. gambiae* s.s. populations under laboratory conditions. This is attributed to similar nutritional elements (Tetramin food), controlled ambient temperature (26°C ± 1) and similar rearing conditions provided in the laboratory for the two populations.

On one hand, during the dry season, the larval development time was shorter in the field experiments compared to the laboratory in the Kayonjo site. Increasing temperatures have been associated with faster larval developmental time (Bayoh & Lindsay, 2003; Bayoh & Lindsay, 2004; Kirby & Lindsay, 2009). Higher temperatures could likely have contributed to the faster larval development time at Kayonjo in the field during the dry period. Indeed Kayonjo site experienced temperatures as high as 34°C during certain periods of the day in the dry season. However, the pupation rate, larval survivorship and pupal weight were not significantly different during the dry season between laboratory and field settings in the Kayonjo site. This finding is in agreement with studies done on *An. gambiae* s.s. in Tanzania (Eliningaya et al., 2005) that showed a similar pupation rate between semi‐field studies and the insectary. On the other hand, during the wet season, semi‐field studies in the Kayonjo site showed that larval development time was much longer than in the laboratory. Bayoh and Lindsay (2003) observed that overall, the rate of development for each *An. gambiae* s.s. immature stage increased at higher temperatures. The lower average temperatures (23°C) of water recorded in these semi‐field habitats during the wet period could partly have contributed to longer larval development and consequently lower pupation rate and pupal weight in semi‐field experiments. These findings are important in determining the timing of the application of biological‐control interventions, which should be designed to coincide with an active larval growth window. In the current study, larval survivorship was similar in the laboratory and semi‐field habitats during the wet season. The observed similarity in survival in the semi‐field experiments during the study could be due to the absence of harsh conditions like heavy rains and predators in the semi‐field habitats. Field data for Kayonjo show that larval developmental time was much longer and larval survivorship lower during the wet season as compared to dry season‐reared larvae. Larvae survivorship and development rely much on temperature, food quality and its availability and light intensity (Kaufman et al., 2006; Rejmankova et al., 2000). It is possible that some of these factors were sub‐optimal during the wet season. For both seasons, more mortality was recorded in second instar larvae hence this is the weakest link in the cycle, and therefore, control interventions need to take this into perspective during design and evaluation.

The pupation rates mirrored trends seen in larval development above in that they were higher during the dry season than in the wet season for the Kayonjo site. This could be partly because, during the dry season, water temperatures tend to rise during certain periods of the day, contributing to faster growth (Kirby & Lindsay, 2009). Higher pupation rates observed in this study could have a fitness advantage for emerging adults and subsequent biological life stages, including avoiding predators in natural populations as previously stated by Kija et al. (2005).

Larval development time in the field during the dry season at the Kibbuye study site was significantly longer within a season and across seasons compared to the Kayonjo site. The Kibbuye site experienced lower habitat temperatures at 19.1°C and 20.3°C compared to the Kayonjo site at 22.7°C and 23°C during the wet and dry season, respectively. The pupation rate at the Kibbuye study site was significantly lower than in the Kayonjo site during the wet season. The larval survivorship at the Kibbuye study site was also much lower than that of the Kayonjo site during the wet season. These variations in life‐history attributes within the season and between seasons suggest complex differences in the ecological factors in mosquito habitats. These findings further emphasize the importance of area‐specific baseline studies over longer periods to inform the control of *An gambiae* populations relating to mosquito release at a particular site.

Fitness costs associated with insecticide resistance can influence mosquito development Rivero et al. (2010), by causing negative effects in mosquito development, reproductive aspects and vector competence, thereby, affecting the vectorial capacity of malaria vectors. Indeed delayed development and reduced survivorship of *An. gambiae* larvae have been reported in western Kenya Osoro et al. (2021). Insecticide resistance may vary between sites, with the Kibbuye study site being more affected in the developmental characteristics of immature *An. gambiae s. s*. than the Kayonjo site.

Water physico‐chemical parameters are weakly correlated with the attributes at the time of measurement. However, water chemical parameters change over time and may affect the number of larvae in the habitat. Our study period may not have been long enough to conclusively detect any effects. There is still a need to understand the tolerance and influence of physico‐chemical parameters on life‐history attributes to come up with more conclusive baseline information for the geographical area.

In conclusion, *An. gambiae* s.s. populations exhibit a difference in life‐history attributes between sites, seasons and in laboratory and field experiments. Temperature and ecological factors could be responsible for differences between laboratory and field data. The knowledge obtained in this study illustrates the importance of life‐history attributes in aiding in the design of disease control interventions, especially to determine appropriate timing, scope and scale of the intervention and amount of control resource allocation. Evaluating the efficacy of various vector control measures, such as the use of genetically modified mosquitoes can be supported. In addition, larval source management methods require information about survivorship and development time.

Studies of life‐history attributes in the field to other localities of Uganda will be of importance for comparing different sites. Life‐history attributes of *An. funestus* and *An. arabiensis* could also be a vital source of information since these are considered important secondary vectors for malaria transmission in the region.

Consent for publication: The authors have agreed to submit the manuscript in its current form for consideration for publication in the Journal.

## CONFLICT OF INTEREST

The author declares that there are no competing interests.

## AUTHOR CONTRIBUTIONS

Charles Batume drafted the first draft of the manuscript and conducted the experiments, Anne M. Akol and Louis G. Mukwaya designed the concept and revised the manuscript critically for important intellectual content. Josephine Birungi revised the manuscript critically for important intellectual content and Jonathan K. Kayondo participated in the design and coordination of the studies, helped to draft and reviewed the manuscript. All authors read and approved the final manuscript.

## Data Availability

The data that support the findings of this study are available from the corresponding author upon reasonable request.
